# The effect of *N*-acetylcysteine on mechanical fatigue resistance of antibiotic-loaded bone cement

**DOI:** 10.1186/s13018-018-0843-9

**Published:** 2018-05-31

**Authors:** Erhan Sukur, Abdulhalim Akar, Huseyin Nevzat Topcu, Ozgur Cicekli, Alauddin Kochai, Mehmet Turker

**Affiliations:** 1 Department of Orthopedics and Traumatology, Sakarya University Research and Training Hospital, 54050 Sakarya, Turkey; 2 Department of Orthopedics and Traumatology, Kocaeli Medical Park Hospital, Kocaeli, Turkey

**Keywords:** *N*-acetylcysteine, Antibiotic-loaded, Bone cement, Peri-prosthetic joint infection

## Abstract

**Background:**

This biomechanical study evaluates the effect of *N*-acetylcysteine alone and in combination with the most commonly used antibiotic-loaded bone cement mixtures.

**Methods:**

We mixed eight bone cement mixture groups including combinations of *N*-acetylcysteine, gentamicin, teicoplanin, and vancomycin and applied a four-point bending test individually to each sample on days 1 and 15 using an MTS Acumen test device.

**Results:**

The result was less than 50 MPa—the limit declared by the ISO (International Standards Organization)—in only the “gentamicin + bone cement + *N*-acetylcysteine” group. Mechanical fatigue resistance of the bone cement decreased significantly with the addition of *N*-acetylcysteine both on day 1 and day 15 (*p* <  0.001). With the addition of *N*-acetylcysteine into the “gentamicin + bone cement” and “vancomycin + bone cement” mixtures, a significant decrease in mechanical fatigue resistance was observed both on day 1 and day 15 (*p* <  0.001). In contrast, with the addition of *N*-acetylcysteine into the “teicoplanin + bone cement” mixture, no significant difference in mechanical fatigue resistance was observed on days 1 and 15 (*p* = 0.093, *p* = 0.356).

**Conclusion:**

Preliminary results indicate that adding *N*-acetylcysteine to teicoplanin-loaded bone cement does not significantly affect the cement’s mechanical resistance, potentially leading to a new avenue for preventing and treating peri-prosthetic joint infection. *N*-acetylcysteine may, therefore, be considered as an alternative agent to be added to antibiotic-loaded bone cement mixtures used in the prevention of peri-prosthetic joint infection.

## Background

Peri-prosthetic joint infection (PJI) is one of the most feared and devastating complications following total joint arthroplasty (TJA). PJI treatment through antibiotics is complex and challenging on account of biofilm formation, which tends to protect pathogens from the effects of systemic antibiotics as well as host immune system [1].

Antibiotic-loaded bone cement (ALBC) prophylaxis promises to be an effective strategy towards reducing the risk of infection following TJA [2] and is commonly used for high-dose local delivery of antibiotics to the surgical site to inhibit biofilm formation and thereby avoiding the occurrence of systemic side effects caused by an over-dosage of antibiotics [1, 3]. For the prophylactic use of ALBC, the antibiotic should not be used at high doses because increasing quantities of antibiotic powder may reduce the compressive and tensile strengths of bone cement [1]. As a result, the amount of antibiotic that can be added to bone cement is limited, thereby limiting their effectiveness against certain micro-organisms. Moreover, adding antibiotics to ALBC may theoretically contribute to an increased resistance towards antibiotics; as a consequence, the probability of obtaining a negative culture result in subsequent aspirations also increases [3, 4].

*N*-acetylcysteine (NAC) is a non-antibiotic drug and antioxidant amino acid that is generally safe and well-tolerated, even at high doses; further, it has a highly favorable risk/benefit ratio and a low rate of adverse events [5, 6]. Studies have shown that NAC reduces biofilm formation, inhibits bacterial adherence, and decreases the production of extracellular polysaccharide matrix and cell viability [7–10]. Since NAC exhibits a synergistic antibacterial and anti-biofilm activity, the proposed study has been designed to evaluate its effects on the mechanical properties of bone cement when used exclusively as well as in combination with commonly used antibiotic mixtures, such as gentamicin, teicoplanin, and vancomycin. It has been hypothesized that its addition would cast a curtailing effect on the biomechanical properties of bone cement, and its combination with antibiotics causes further aggravation of this effect.

## Methods

### Cement mixture preparation

Eight bone cement mixture groups were prepared containing various combinations of bone cement, NAC along with varying concentrations of gentamicin, teicoplanin, and vancomycin, as described in Table 1. Each group was prepared by mixing the ingredients together for 45 s using commercially available mechanical mixing bowls under a constant vacuum pressure of approximately 200 mbar in a controlled environment with temperature and relative humidity values corresponding to 22 ± 1 °C and 40–60%, respectively [11].

**Table 1:** Cement mixture groups

Group	Cement mixture ingredients
1	Bone cement^a^
2	Bone cement + 600 mg NAC^b^
3	Bone cement + 0.5 g gentamicin^c^
4	Bone cement + 0.5 gentamicin + 600 mg NAC
5	Bone cement + 1 g vancomycin^d^
6	Bone cement + 1 g vancomycin + 600 mg NAC
7	Bone cement + 400 mg teicoplanin^e^
8	Bone cement + 400 mg teicoplanin + 600 mg NAC

^a^Versabond cement (40 g polymer powder + 20 mL monomer liquid)

^b^Acetyl-l-cysteine (Sigma Aldrich, A7250)

^c^Cemex Genta

^d^Edicin 1 g (Sandoz, Serbia)

^e^Targocid, Sanofi Aventis, Italy

### Sample preparation

**Fig. 1:**
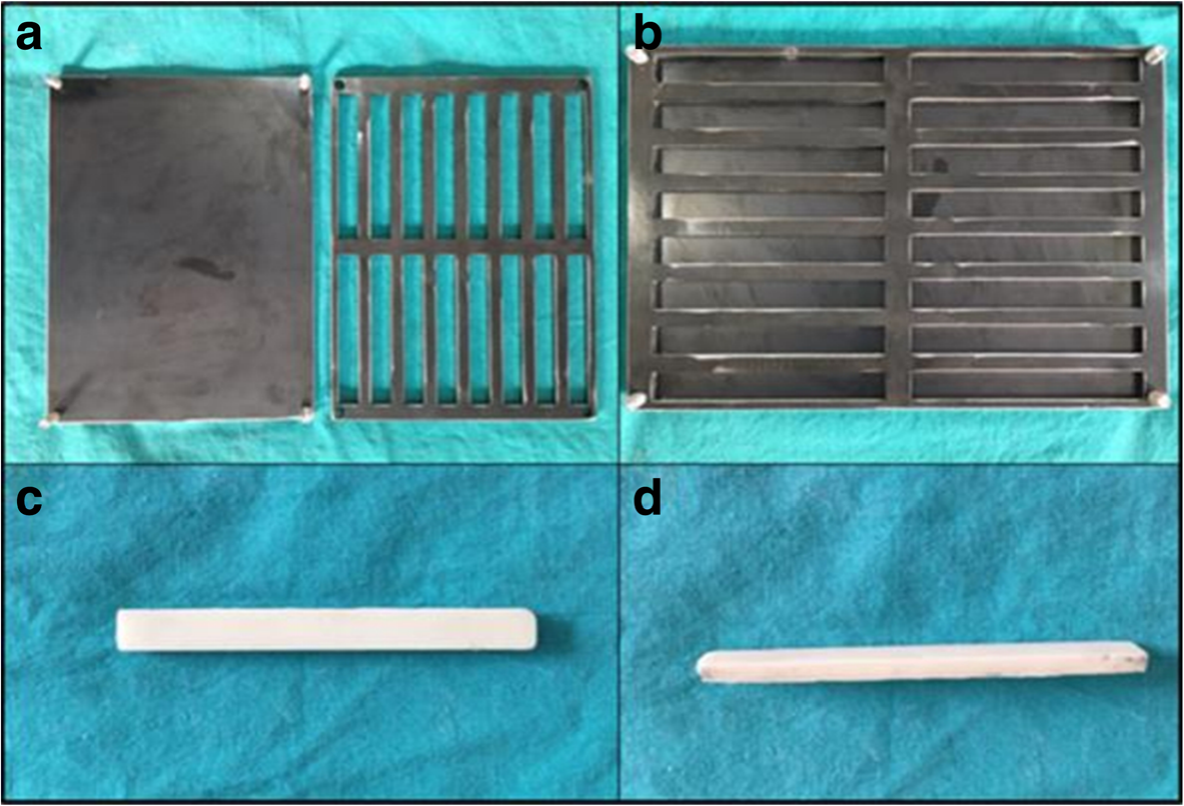
**a**–**d** Rectangular-prism-shaped molds

Special rectangular-prism-shaped molds measuring 3.3 × 10 × 75 mm, as shown in Fig. 1a, b), were prepared in accordance with the size recommended by the International Standards Organization (ISO 5833). Upon attainment of dough-like viscosity values, the prepared mixtures were made to fill the prism-shaped molds by pouring them into the molds simultaneously by hand and applying pressure to completely occupy all molds and gaps. Subsequently, the molds were pressed between two metal plates through use of a clamp and maintained between the plates for 15 min to facilitate cement-mixture hardening. Burrs, created during removal of mixture samples from the molds using a special remover, were subsequently cleaned. The samples were then macroscopically examined for manufacturing defects (as depicted in Fig. 1c, d). Defected samples, wherein more than 10% of the section surface exhibited signs of defects, were discarded from the study. Twenty samples were prepared from each group, 10 of which were evaluated on day 1 while the remaining were evaluated on day 15. Samples scheduled for evaluation on day 15 were set apart for 15 days to be maintained in a 37 °C normal saline bath, wherein antibiotics were released from the surface of the sample. In order to prevent saturation of the environment due to antibiotic release and facilitate maximum antibiotic secretion, the saline solution containing the samples was changed on a daily basis.

### Biomechanical testing

**Fig. 2:**
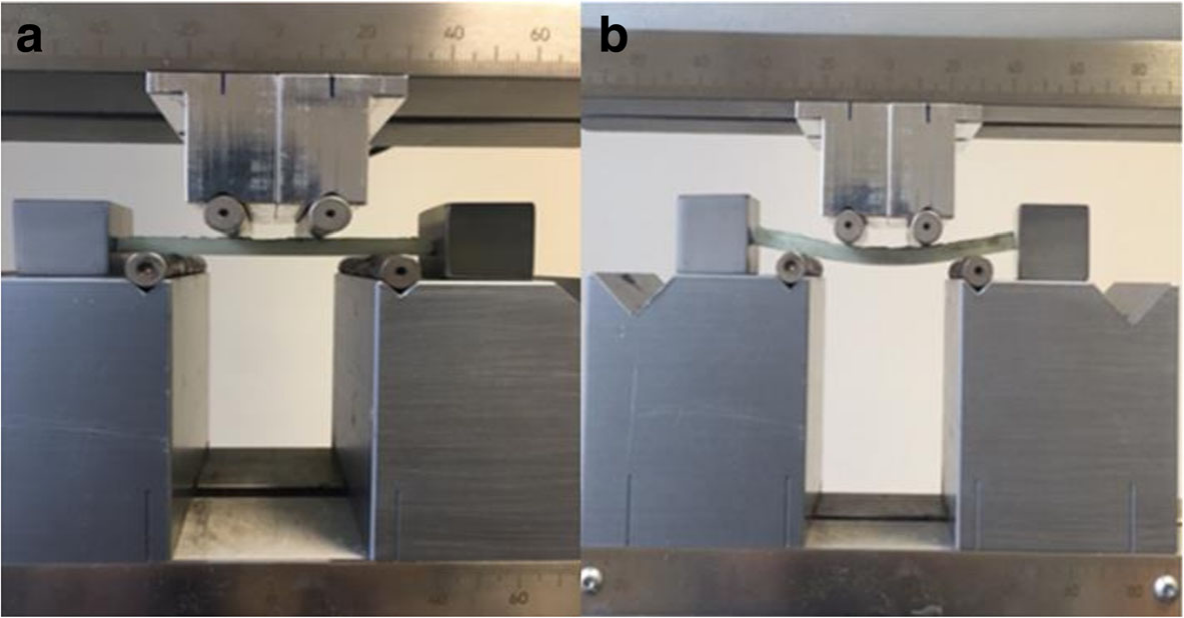
**a**, **b** Demonstration of four-point bending test

Acumen (MTS Systems Corporation, MN, USA) electrodynamic testing device was used for biomechanical evaluation of the prepared samples. A four-point bending test was independently performed on 10 samples each on day 1 and day 15. During the test, a certain bending force was exerted upon the samples at a speed of 3 mm/min, and corresponding uniaxial bending stress values were calculated in MPa (*N*/mm2). The value of the bending stress recorded at the instant at which the sample failed was considered as the mechanical fatigue resistance limit (Fig. 2a, b).

### Statistical analysis

Statistical evaluation in the proposed study was performed using the SPSS 22.0 application for Windows (SPSS Inc., Chicago, IL, USA). Descriptive statistics were provided to represent the mean and standard deviation for numeric variables. Student’s *t* test was performed when numeric variables were normally distributed, and multiple groups were compared using the one-way ANOVA test. Additionally, the Mann–Whitney *U* test and the Kruskal–Wallis test were performed when numeric variables did not exhibit normal distribution. For a parametric sub-group analysis comprising more than two groups, the Tukey test was performed. For non-parametric analyses, on the other hand, the Mann–Whitney *U* test with Bonferroni correction was preferred. The significance level for each of the above tests was set at 0.05.

## Results

**Fig. 3:**
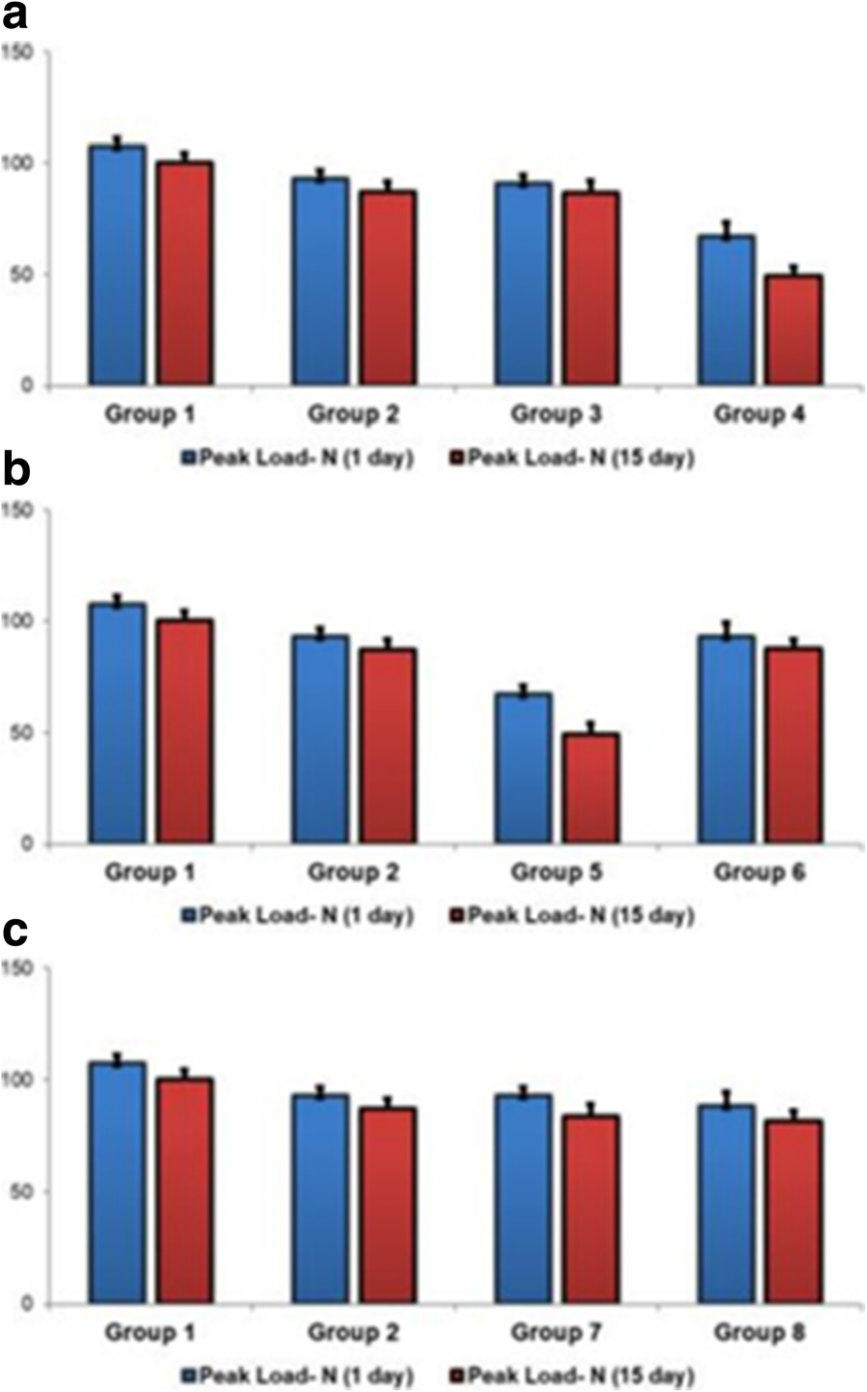
**a**–**c** The comparison of the results

Results of the biomechanical evaluation described above are summarized and presented in Table 2. As seen in Table 2, only samples belonging to group 4 demonstrated a value of the failure bending stress less than 50 MPa (the limiting value declared by ISO). The mechanical fatigue resistance (MFR) of bone cement samples was found to have significantly decreased upon addition of NAC. This result was consistent in the biomechanical testing of samples performed on day 1 as well as day 15 (*p* <  0.001). As depicted in Fig. 3a, NAC addition to the group 3 mixture causes a significant decrease in MFR (*p* <  0.001), as observed on day 1 as well as day 15. In the case of NAC addition to the group 5 mixture, a significant difference was observed between MFR values recorded on day 1 and day 15 (*p* <  0.001), as depicted in Fig. 3b. NAC addition to the group 7 mixture, however, demonstrated no significant difference between MFR values recorded on day 1 and day 15 (*p* = 0.093 and *p* = 0.356, respectively), as depicted in Fig. 3c.

**Table 2:** Results of the biomechanical tests

	Peak load *N* (day 1)	Peak load *N* (day 15)	
	Mean ± SD	Mean ± SD	*p*
Group 1	107.7 ± 3.5	100.4 ± 4.1	0.001
Group 2	93.3 ± 3.4	87.3 ± 4.6	0.017
*p*	< 0.001	< 0.001	
Group 3	91.2 ± 3.5	86.8 ± 5.1	0.005
Group 4	77.3 ± 6.3	49.2 ± 4.2	< 0.001
*p*	< 0.001	< 0.001	
Group 5	93.2 ± 6.1	87.6 ± 3.2	0.077
Group 6	76.3 ± 7.6	62.4 ± 3.3	0.002
*p*	< 0.001	< 0.001	
Group 7	93.2 ± 4.3	83.9 ± 4.0	< 0.001
Group 8	88.5 ± 2.8	81.9 ± 4.4	0.001
*p*	0.093	0.356

## Discussion

Although some in vitro studies have evaluated the antibiofilm and antimicrobial effects of NAC in bone cement, to our knowledge, this is the first study to evaluate the biomechanical features of NAC and ALBC applied together. We observed that adding NAC to teicoplanin-loaded bone cement did not affect its MFR on day 1 or day 15. On the other hand, adding NAC into gentamicin- and vancomycin-loaded bone cement decreased biomechanical features of the bone cement. Biofilm formation is a bacterial behavior that impedes the effectiveness of PJI therapy [5], and inadequate ALBC application may lead to the proliferation of resistant bacteria strains and cannot obviate biofilm formation [12]. In our study, we have chosen to evaluate a combination of ALBC and NAC, because NAC is a new pharmacological approach to inhibiting biofilm formation, eradicating mature biofilms and increasing the permeability of antibiotics to overcome antibiotic resistance [5]. We expected that adding NAC into ALBC may worsen its mechanical properties by increasing the total number of particles added to the cement. Although compression tests are commonly performed to evaluate the biomechanical resistance of bone cement, we performed four-point bending tests as described by ISO, because they are more sensitive to surface defects and internal porosity [13, 14].

ALBC is used to avoid systemic toxicity from antibiotics and maximize their local concentration, inhibiting biofilm formation at the infection site in the treatment and prophylaxis of PJI [15–17]. Currently, antibiotic doses in ALBC vary depending on the application. When ALBC is used to treat an existing infection, appropriate therapeutic levels can be achieved by loading more than 2 g of antibiotics into 40 g of bone cement [1]. However, the addition of antibiotics into bone cement decreases its compressive and tensile strength. Furthermore, this decrease is augmented by increasing the antibiotic dose [18]. Hence, a maximum of 1–2 g of antibiotics can be loaded into 40 g of bone cement for infection prophylaxis [1, 18, 19].

In the current study, the cement mixtures were formed by adding prophylactic doses of three types of antibiotics (0.5 g gentamicin, 1 g vancomycin, and 400 mg teicoplanin) and 600 mg NAC into bone cement to mimic prophylactic application. Consistent with the literature, we observed a significant decrease in mechanical properties with the addition of antibiotics alone into the bone cement in a four-point bending test [1, 18]. Similarly, adding only NAC negatively affected the bone cement’s mechanical properties. However, we found no available literature on the effect of NAC addition into bone cement on its biomechanical properties. Thus, it was not possible to compare our results. We postulate that adding NAC to bone cement decreased its mechanical properties by increasing the number of particles in the cement.

A homogenous mixing and pressurization during implantation are crucial for better mechanical properties of the bone cement. The use of vacuum-mixing of the cement causes less porosity and less reduction of the tensile fatigue strength of ALBC than hand-mixing [1]. In a current study, it was concluded that the standard method using a plastic pressurizer with cement gun after vacuum mixing appears to be adequate for achievement of optimum pressurization during femoral cementing without increased risk of embolization [19]. In our study, we used the vacuum mixing method to optimize the cement porosity.

Our results showed that 600 mg of NAC may be safely added to bone cement while maintaining sufficient biomechanical resistance against bending and shearing forces. However, in this study, adding NAC to gentamicin-loaded bone cement significantly decreased the mechanical strength of the mixture, consistent with our hypothesis. Our four-point bending test result was less than the minimum ISO requirement of 50 MPa in the *0.5 gentamicin + 600 mg NAC + bone cement* group. Similarly, Lautenschlager et al. observed decreased mechanical resistance in gentamicin-loaded bone cement [20]. In our study, adding NAC to vancomycin-loaded bone cement significantly decreased its biomechanical resistance; however, this mixture achieved bending test results of at least 50 MPa on both days 1 and 15. By contrast, adding NAC to 400 mg teicoplanin-loaded bone cement did not adversely affect its mechanical properties. Gogus et al. investigated the effects of increasing doses of teicoplanin in bone cement on the cement’s biomechanical properties and found that doses greater than 2000 mg added into bone cement (40 g polymer powder + 20 mL monomer liquid) decreased its mechanical resistance past the critical lower limit. In addition to the concentration and type of antibiotics, the brand of the antibiotic and the bone cement also affects biomechanical properties [20–22], which might cause the variability of the results in the available literature.

The main limitation of our study was that the antibiotics were added to the bone cement only in prophylactic doses, and the effect of only a single dose of NAC was evaluated. Determining the correct dose of NAC for use in combination with optimized antibiotic doses in bone cement requires further study. Additionally, only a four-point bending test was performed; compressive resistance limits were not evaluated in our study.

## Conclusions

This study examines the mechanical properties of antibiotic-loaded bone cement after the addition of *N*-acetylcysteine. We have shown preliminary results indicating that adding *N*-acetylcysteine to teicoplanin-loaded bone cement does not significantly affect the cement’s mechanical resistance, potentially leading to a new avenue for preventing and treating PJI. However, this effect varies with the type and amount of antibiotic used in the mixtures. Bone cement mixtures containing a combination of NAC and antibiotics may be manufactured and used clinically.

## Abbreviations

ALBC: Antibiotic-loaded bone cement
ANOVA: Analysis of variance
ISO: International Standards Organization
MFR: Mechanical fatigue resistance
NAC: *N*-acetylcysteine
PJI: Peri-prosthetic joint infection
TJA: Total joint arthroplasty
